# In-hospital mortality risk prediction models for patients with acute coronary syndrome: a systematic review and meta-analysis

**DOI:** 10.3389/fcvm.2025.1659184

**Published:** 2025-10-31

**Authors:** Rui Jian, Jie Zhang, Yuxiu Zeng, Tian Zhou, Yan Wu, Lewen Wu, Yang Yu, Chongcheng Xi

**Affiliations:** ^1^ Chengdu University of Traditional Chinese Medicine, Chengdu, China; ^2^ Chongqing Health College, Chongqing, China

**Keywords:** models, acute coronary syndrome, in-hospital mortality, risk prediction, systematic review

## Abstract

**Objective:**

To systematically evaluate in-hospital mortality risk prediction models for patients with acute coronary syndrome (ACS) and provide valuable insights and references for the construction, application, and optimization of these models.

**Methods:**

A comprehensive search was conducted in five databases, including CNKI, Wanfang, PubMed, Web of Science, and Embase, from inception to November 2024. Researchers screened the literature, extracted relevant data, and assessed the quality of the prediction models using the Prediction Model Risk of Bias Assessment Tool (PROBAST). Extracted data included study design, data sources, outcome definitions, sample size, predictive factors, model development, and performance.

**Results:**

A total of 18 studies involving 44 prediction models were included. The area under the receiver operating characteristic curve (AUC) or C-index of these models ranged from 0.79 to 0.96. Overall, the included prediction models demonstrated a high risk of bias, primarily due to issues such as unreported missing data, methodological flaws in model construction, and a lack of model performance evaluation.

**Conclusion:**

The construction of in-hospital mortality risk prediction models for patients with ACS is still in the developmental stage. Future development and validation of prediction models should adhere to the PROBAST and TRIPOD guidelines to establish models with strong predictive performance and high generalizability.

**Systematic Review Registration:**

PROSPERO CRD42024567755.

## 1 Background

Acute coronary syndrome (ACS) is a severe cardiovascular condition that encompasses three clinical types: ST-segment elevation myocardial infarction (STEMI), non-ST-segment elevation myocardial infarction (NSTEMI), and unstable angina (UAP). Globally, more than 7 million people are diagnosed with ACS each year. In 2023, approximately 50% of cardiovascular-related deaths were attributable to this condition, underscoring ACS as one of the leading causes of mortality worldwide (1, 2). During hospitalization, more than 5% of ACS patients experience in-hospital mortality, with certain subgroups showing mortality rates as high as 26.7%. Long-term follow-up studies indicate mortality rates reaching up to 26.5%. In contemporary cohorts of STEMI patients, in-hospital mortality exceeds 50% (3, 4). Additionally, a Swiss study reported that in-hospital mortality rates significantly increase when ACS is accompanied by multivessel disease, with no observed improvement in this trend over time (5). Therefore, accurately identifying the in-hospital mortality risk in ACS patients is crucial for developing effective treatment strategies. In recent years, numerous prediction models for in-hospital mortality risk in ACS patients have emerged (6). Among them, the Global Registry of Acute Coronary Events (GRACE) score and the Thrombolysis in Myocardial Infarction (TIMI) score are the most recommended and widely used risk assessment tools in clinical practice guidelines. Additionally, the Acute Coronary Treatment and Intervention Outcomes Network (ACTION) risk model has demonstrated excellent performance in predicting in-hospital mortality (7). However, a systematic evaluation of the quality and applicability of these models in different clinical settings remains lacking. This study aims to systematically review and evaluate existing in-hospital mortality risk prediction models developed for ACS patients, providing valuable references for scholars in the construction, optimization, and validation of such models. The findings of this study will offer critical scientific support for clinical practice and future research.

## 2 Methods

The study protocol was registered on PROSPERO (Registration Number: CRD42024567755).

### 2.1 Search strategy

To ensure a comprehensive literature search and account for the broad dissemination of relevant studies, both Chinese and English databases were searched. The databases included CNKI, Wanfang, PubMed, Embase, and Web of Science, with the search period extending from the inception of each database to November 2024. A combination of subject terms and free-text terms was used in both Chinese and English searches. The main Chinese and English search terms are acute coronary syndrome (ACS), including its various variant forms such as acute coronary syndromes (plural form), coronary syndrome, acute (inverted form), and coronary syndromes, acute (inverted plural form); patients with acute coronary syndrome; risk, risk assessment, relative risk; death, cardiac death, in-hospital death, mortality rate, in-hospital mortality rate, risk of death; prediction, early warning, influencing factors, impact factor; risk prediction, model, tool and score. The detailed search strategy is provided in the supplementary material. To further enhance the comprehensiveness and accuracy of the literature collection, manual searching and a snowballing method were employed to supplement the references and citations of the included studies.

### 2.2 Inclusion and exclusion criteria

Inclusion Criteria: (1) Population: Patients with acute coronary syndrome (ACS), including ST-segment elevation myocardial infarction (STEMI), non-ST-segment elevation myocardial infarction (NSTEMI), and unstable angina (UAP). (2) Study Focus: Construction or validation of in-hospital mortality risk prediction models for ACS patients. (3)Study Design: Observational studies.

Exclusion Criteria: (1) Non-English and non-Chinese publications. (2) Duplicate studies and articles with inaccessible full texts. (3) Publications in the form of abstracts, conference notices, reviews, or meta-analyses. (4) Studies that only analyzed predictive factors for in-hospital mortality in ACS patients without constructing a prediction model.

### 2.3 Literature screening and data extraction

Two researchers independently screened the literature by reviewing the titles and abstracts according to the inclusion and exclusion criteria. In cases of disagreement, discussions or consultation with a third party were conducted to reach a consensus. After excluding irrelevant studies, the full texts of the remaining articles were thoroughly reviewed to determine the final included studies. Data extraction was guided by the Critical Appraisal and Data Extraction for Systematic Reviews of Prediction Modelling Studies (CHARMS) checklist (8). Extracted data included: Publication Date, Study Design, Country, Data Source, Sample Size, Candidate Variables, Modeling Methods, Variable Selection Methods, Number of Models, Model Performance, Model Validation Methods, Model Presentation Format.

### 2.4 Risk of bias and applicability assessment

Two researchers independently assessed the risk of bias and applicability of the included studies using the Prediction Model Risk Of Bias Assessment Tool (PROBAST) (9). In case of disagreement, a third party's opinion was sought.

The risk of bias assessment covered four domains: participants, predictors, outcomes, and analysis, comprising a total of 20 specific questions. Following the “shortest plank theory,” each domain was evaluated as follows: Low Risk:If all items were marked as “probably yes” or “yes.” High Risk: If any item was marked as “no” or “probably no.” Unclear Risk: If insufficient information was provided for any item. For the overall risk of bias, a study was classified as “low risk” only if all four domains were rated as “low risk.” If any domain was rated as “high risk,” the overall bias risk was deemed “high.” If any domain was rated as “unclear,” the overall risk of bias was also classified as “unclear.” Applicability was evaluated across three domains: study population, predictors, and outcomes. The assessment method was consistent with the risk of bias evaluation, using the same criteria for low, high, and unclear applicability.

### 2.5 Data synthesis

A descriptive analysis method will be used to summarize the basic characteristics of the included studies and the constructed prediction models.

## 3 Results

### 3.1 Literature screening process and results

**Figure 1:**
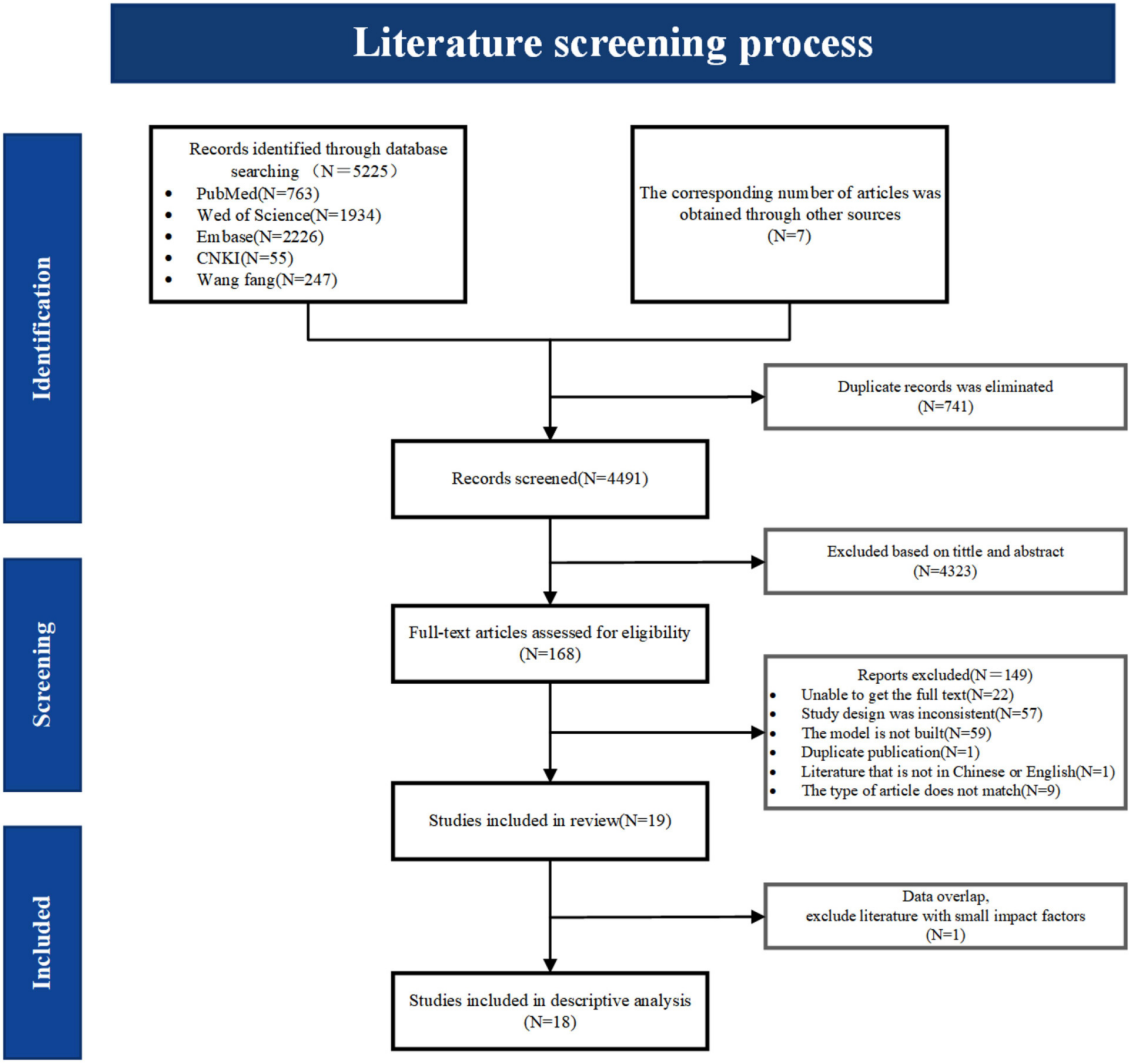
Literature screening process diagram. During the data analysis, two included literatures showed overlapping data in terms of modeling methods, data time, and screening variables. To avoid bias in the analysis results, the one with a lower impact factor was excluded.

A total of 5,232 relevant studies were identified through database searches and other resources. After removing 741 duplicate records, 4,491 articles remained. Initial screening of titles and abstracts resulted in 168 studies for full-text review. Following a detailed assessment, 19 studies were deemed eligible, and finally, 18 studies were included in the descriptive analysis. The screening process is shown in Figure 1.

### 3.2 Basic characteristics of included studies

This study conducted a comprehensive analysis of 18 relevant studies (10–27). It was found that 67% (12/18) of the in-hospital risk prediction models for acute coronary syndrome (ACS) were published within the past five years (10–15, 18, 19, 21, 23, 26, 27). These studies primarily originated from China (*n* = 9) (9, 10, 11, 13, 19, 20, 23, 25–27), the United States (*n* = 2) (12, 21), and Poland (*n* = 2) (16, 24). All included studies were retrospective cohort studies. In terms of data sources, half of the studies were based on single-center data (10, 11, 13, 15–18, 23, 24), while the other half utilized multi-center data (12, 14, 19–22, 25–27). All studies reported the sample sizes required for model development, with sample sizes ranging from 502 to 755,402. The in-hospital mortality rates varied between 1.88% and 12.3%. For details, please refer to Table 1.

**Table 1:** Basic characteristics of included literature.

First author/year of publication	Area	Research design	Data sources	Model type	Research object	Final result	Total sample		
							Number of events	Total number of people	In-hospital mortality rate
Jun Ke (10)/2022	China	①	Single-center	^b^	ACS	A	122	6,482	1.88%
Rong Li (11)/2023	China	①	Single-center	^b^	ACS	A	85	2,414	3.5%
Ashraf Abugroun (12)/2020	America	①	Multicenter	^b^	ACS undergoing PCI	A	6,312	252,443	2.5%
Bai Li (13)/2023	China	①	Single-center	^a^	ACS undergoing CAG or PCI	B	414	19,237	2.2%
Sazzli Kasim (14)/2022	Malaysia	①	Multicenter	^b^	STEMI and NSTEMI	A	4,809	68,528	7.02%
Claudio Parco (15)/2021	Germany	①	Single-center	^a^	STEMI and NSTEMI	B	119	1,567	7.5%
Konrad Pieszko (16)/2018	Poland	①	Single-center	^b^	ACS	A	97	6,769	1.4%
Raposeiras-Roubin S (17)/2012	Spain	①	Single-center	^a^	ACS	A	265	4,497	5.9%
Raymond Bernardus (18)/2023	Indonesia	①	Single-center	^a^	ACS	A	159	1,504	10.6%
Qiang Chen (19)/2022	China	①	Multicenter	^a^	AMI	A	40	613	6.5%
Rui Fu (20)/2018	China	①	Multicenter	^b^	NSTEMI	A	342	5,775	5.92%
Rohan Khera (21)/2021	America	①	Multicenter	^b^	AMI	A	33,238	755,402	4.4%
Joon-Myoung Kwon (22)/2019	Korea	①	Multicenter	^b^	AMI	A	1,081	22,875	4.4%
He Lin (23)/2024	China	①	Single-center	^b^	Elderly patients with AMI	A	62	502	12.3%
Konrad Pieszko (24)/2019	Poland	①	Single-center	^b^	ACS	A	83	5,053	1.64%
Chenxi Song (25)/2018	China	①	Multicenter	^b^	AMI	A	1,504	23,417	6.4%
Jingang Yang (26)/2024	China	①	Multicenter	^b^	AMI	A	2,416	30,849	7.8%
Peng Ran (27)/2021	China	①	Multicenter	^b^	ACS	A	1,181	62,546	1.9%

Special symbols are used to represent the corresponding content due to its length. ①: retrospective cohort study.

^a^ Verification.

^b^ Development and validation; A: In-hospital death; B died in the backyard after surgery; ACS, acute coronary syndrome; AMI, acute myocardial infarction; STEMI, ST segment elevation myocardial infarction; NSTEMI, non ST segment elevation myocardial infarction; PCI, percutaneous coronary intervention therapy; CAG, coronary angiography.

### 3.3 Basic information on prediction model construction

The number of candidate variables varied widely among studies, ranging from 8 to 89. When handling continuous variables, the majority of the studies retained their continuous nature, with only two studies converting continuous variables into binary categories (18, 20). For missing data, common approaches included exclusion and imputation. The variable selection process typically followed a stepwise procedure, starting with univariate analysis and subsequently proceeding to multivariate analysis. In terms of modeling methods, most studies employed traditional regression analysis to construct models, while others integrated machine learning or deep learning techniques. For specifics, please refer to Table 2.

**Table 2:** Basic information for constructing prediction models.

Author	Candidate variables		Missing data		Variable selection	Modeling method
	Number	Continuous variable method	Number	Processing method		
Jun Ke (10)/2022	22	MC	No	Mean Imp	UMA	ML:LR, GBDT, RF, SVM
Rong Li (11)/2023	44	MC	3,585	Imp	UMA	LR, XGBoost
Ashraf Abugroun (12)/2020	8	MC	No	Exclude	UMA	LR
Bai Li (13)/2023	24	MC	3,964	MF Imp	No	No
Sazzli Kasim (14)/2022	54	MC	55,338	MF Imp, Multi Imp	FSA	ML:LR, RF, SVM;DL:LR, RF, SVM
Claudio Parco (15)/2021	No	MC	1,510	Imp	No	No
Konrad Pieszko (16)/2018	23	MC	No	Mean Imp, VC-DomLEM Imp	UA	LR, XGBoost, DRSA-BRE
Raposeiras-Roubin S (17)/2012	13	MC	107	Exclude	No	No
Raymond Bernardus (18)/2023	14	Bin Var	No	No	MA	LR
Qiang Chen (19)/2022	No	MC	No	Exclude	UMA	LR
Rui Fu (20)/2018	21	Bin Var	393	Mean Imp or Med Imp	UMA	LR
Rohan Khera (21)/2021	56	MC	295,987	Mode Imp, 5× MI	No	LR, LASSO, XGBoost, Neural net
Joon-Myoung Kwon (22)/2019	No	MC	3,102	Exclude	No	DL, ML:LR, RF
He Lin (23)/2024	26	MC	No	MI	UA, LASSO Reg	COX
Konrad Pieszko (24)/2019	28	MC	394	Exclude	UMCA	COX, XGBoost
Chenxi Song (25)/2018	25	MC	2,619	Exclude	UMA	LR
Jingang Yang (26)/2024	89	MC	Provide missing rate	MICE	ML	XGBoost
Peng Ran (27)/2021	32	MC	1,095	Exclude	UMA	LR

MC, maintain continuity; Bin Var, binary variable; Mean Imp, Fill in the average value; Imp, fill; MF Imp, Massforest algorithm filling; Multi Imp, multivariate filling; VC DomLEM Imp, VC DomLEM algorithm filling; Med Imp, median filling; Mode Imp, Pattern filling; 5×MI, 5× multiple filling; MI, multiple filling; MICE, chain equation multiple interpolation; UMA, single factor analysis followed by multiple factor analysis; UMCA, single factor analysis followed by COX regression analysis; FSA, feature selection algorithm; UA, univariate analysis; MA, multivariate analysis; LASSO Reg, LASSO regression; ML, machine learning; LR logistic regression; GBDT, gradient boosting decision tree; RF, random forest; SVM, support vector machine; XGBoost, gradient boosting tree; DL, deep learning DRSA-BRE, dominated rough set balancing rule ensemble; COX, proportional hazard regression model.

### 3.4 Characteristics of prediction models

#### 3.4.1 Predictive performance of models

All included studies reported discrimination metrics, including the area under the curve (AUC) or C-index, ranging from 0.79 to 0.96. These values indicate that most prediction models demonstrated at least moderate accuracy and good discriminatory ability. In terms of model calibration, the most commonly used test method was the Hosmer-Lemeshow (HL) goodness-of-fit test, which was employed in six studies (13, 17, 20, 25–27), followed by the calibration slope, used in four studies (11, 12, 19, 21). Additionally, five studies did not provide calibration information (10, 16, 18, 22, 24), while others utilized calibration plots, calibration curves, calibration intercepts, nomograms, or the Brier score either individually or in combination.

The results indicated good calibration performance. For details, please refer to Table 3.

**Table 3:** The predictive performance of the prediction model.

Author	Model performance	
	AUC	Calibration
Jun Ke (10)/2022	LR:0.884, XGBoost:0.918, RF:0.913, SVM:0.896	No
Rong Li (11)/2023	LR:0.904, XGBoost:0.913	Calibrate slope, calibration intercept, and Brier score
Ashraf Abugroun (12)/2020	0.83	Calibration slope, calibration intercept, calibration chart
Bai Li (13)/2023	GRACE:0.926, GRACE2.0:0.920, ACTION:0.945, TIMI:0.811, CPACS:0.841	Graphical analysis of risk model calibration/goodness of fit, HL
Sazzli Kasim (14)/2022	Optimization model:0.96	McNemar test, hyperparameter adjustment
Claudio Parco (15)/2021	GRACE 1.0: 0.84; GRACE 2.0: 0.79; ACTION: 0.84; NCDR: 0.89	Calibration chart
Konrad Pieszko (16)/2018	LR: 68 ± 11, XGBoost: 78 ± 3, DRSA-BRE: 80.8	No
Raposeiras-Roubin S (17)/2012	Original GRACE RS: 0.91; Update GRACE RS: 0.90; AR-GRS: 0.90	HL
Raymond Bernardus (18)/2023	0.820	No
Qiang Chen (19)/2022	0.814	Calibration curve, calibration slope, calibration intercept, Brier score
Rui Fu (20)/2018	0.81	HL
Rohan Khera (21)/2021	LR: 0.888, LASSO: 0.886, XGBoost:0.898, Neural net:0.885, meta-classification0.899	Calibrate slope, Brier score, shift schedule
Joon-Myoung Kwon (22)/2019	Optimization mode:STEMI: 0.905; NSTEMI: 0.870	No
He Lin (23)/2024	10 day in-hospital death: 0.9079; 20 day in-hospital death: 0.8355;	Calibration curve
Konrad Pieszko (24)/2019	0.89	No
Chenxi Song (25)/2018	0.83	HL
Jingang Yang (26)/2024	0.896	Calibration chart, HL
Peng Ran (27)/2021	0.84	HL

CPACS, clinical pathway syndrome of acute coronary artery; NCDR, national cardiovascular disease data registry center; AR-GRS, action registry and GWTG (Get with the guidelines) database risk score.

#### 3.4.2 Predictive factors in the models

**Figure 2:**
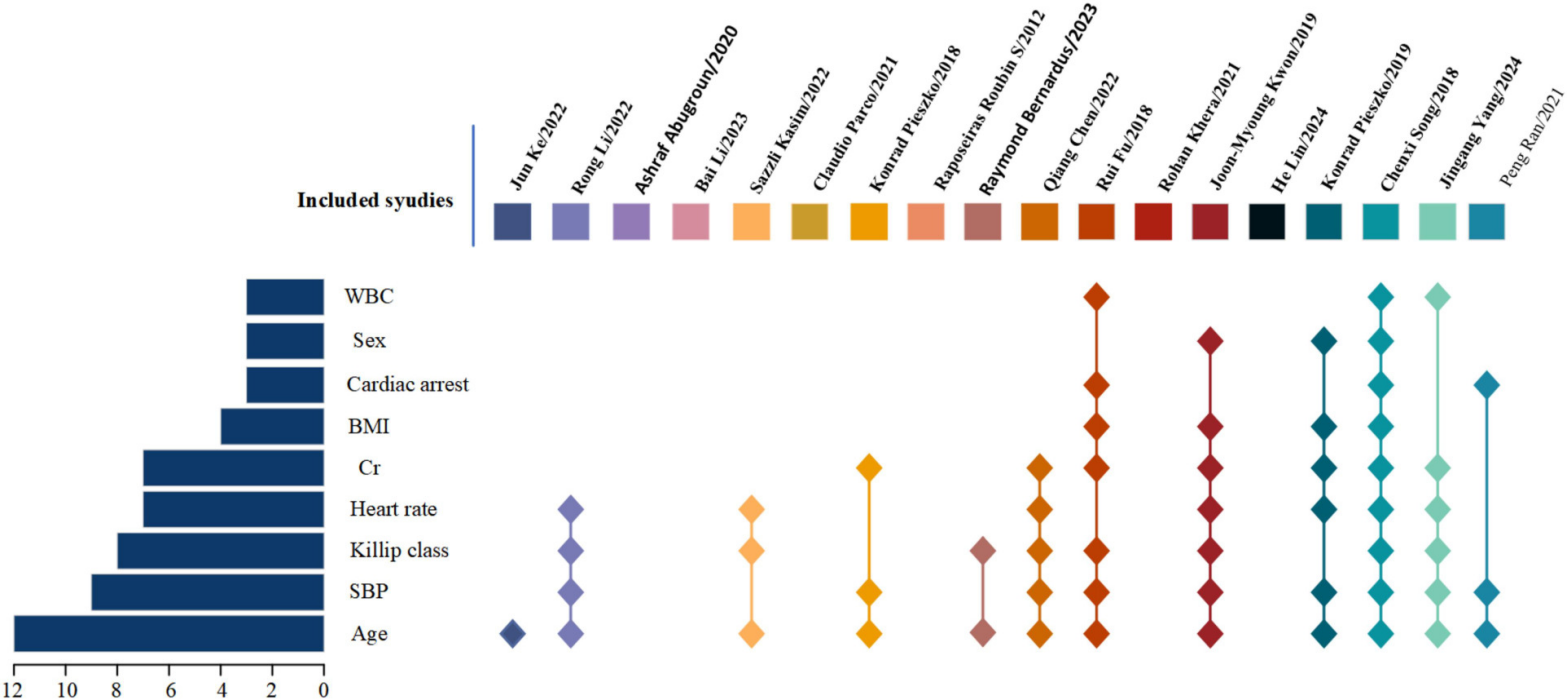
Distribution of occurrence frequencies of common predictors in Included literatures.

The number of predictive factors included in the models ranged from 5 to 20. However, four studies (13, 15, 17, 21) did not provide detailed information about the final predictive factors included in their models. Among the top nine predictors most frequently included in the models, age (*n* = 12), systolic blood pressure (*n* = 9), Killip classification (*n* = 8), heart rate (*n* = 7), creatinine (*n* = 7), body mass index (*n* = 4), cardiac arrest (*n* = 3), sex (*n* = 3), and white blood cell count (*n* = 3) were the most prominent. For details, please refer to Table 4 and Figure 2.

**Table 4:** Prediction factors of the prediction model.

Author	Final predictive factor	
	Number	Content
Jun Ke (10)/2022	10	NT-proBNP, D-dimer, cTnI, age, HDL-C, statins, NSTEMI, Killip III, Killip IV, CK
Rong Li (11)/2023	20	HR, age, MB, LAD, LVEDD, RCA stenosis, BNP, LM stenosis, CK-MB, cTnI, Killip class, renal dysfunction, elevated Cre, elevated MB, history of PCI, presentation in CS, elevated BNP, elevated HR, Higher BMI, SBP
Ashraf Abugroun (12)/2020	7	CHF, Hypotension/CS, Age ≥65, Age ≥75, DM, Stroke, PVD
Bai Li (13)/2023	No	No
Sazzli Kasim (14)/2022	14	Age, HR, Killip class, FBG, anti-arrhythmic agent, LDL, HDL, statins, lipid lowering agent, chronic angina past 2 weeks, ST-segment elevation ≥1 mm in ≥2 contiguous limb leads, CABG, oral hypoglycemic agent, cardiac catheterization
Claudio Parco (15)/2021	No	No
Konrad Pieszko (16)/2018	5	Neutrophil count, SBP, Cr, age, hematocrit
Raposeiras-Roubin S (17)/2012	No	No
Raymond Bernardus (18)/2023	5	Age, history of angina, history of revascularization, modified shock index, Killip class
Qiang Chen (19)/2022	9	Age, HR, SBP, Cr, Killip class, ST-segment deviation, cardiac biomarkers, CA at admission, SHR
Rui Fu (20)/2018	11	Age, BMI, SBP, Killip class, CA, ECG ST-segment depression, Cr, WBC, smoking status, previous MI, previous PCI
Rohan Khera (21)/2021	No	No
Joon-Myoung Kwon (22)/2019	13	Age, sex, BMI, CA before visit, SBP, HR, Killip class, CK-MB, blood glucose, CRP, Cr, LDL, elevation of the ST segment
He Lin (23)/2024	8	Ventricular tachycardia fibrillation, AF, nicorandil, βblockers, ACCI, CO2CP, Ca, ACEI/ARB
Konrad Pieszko (24)/2019	19	troponin elevation ratio, NLR, PLR, RDW, CRP, platelet count, Cr, Hb, MCV, Na, PT, fibrinogen, age, neutrophil count, BMI, SBP, DBP, HR, sex
Chenxi Song (25)/2018	16	Age, sex, BMI, SBP, HR, Cr, WBC, K, Na, ECG ST-segment elevation, anterior wall involvement, CA, Killip class, hypertension, hyperlipidemia, smoking status
Jingang Yang (26)/2024	10	Age, LVEF, Killip class, HR, Cr, blood glucose, WBC, SBP, ACEI/ARB, TC
Peng Ran (27)/2021	7	Age, SBP, CA, ITDM, history of AF, AHF and/or CS, ST-segment deviation

NT proBNP, N-terminal B-type natriuretic peptide precursor; cTnI, troponin I; HDL-C, high density lipoprotein cholesterol; CK, creatine kinase; HR, heart rate; MB, myoglobin; LAD, left atrial diameter; LVEDD, left ventricular end diastolic diameter; RCA, right coronary artery; BNP, brain natriuretic peptide; LM, left main trunk; CK-MB, creatine kinase isoenzyme; CS, cardiogenic shock; BMI, body mass index; SBP, systolic blood pressure; CHF, congestive heart failure; DM, diabetes; PVD, peripheral vascular disease; FBG, fasting blood glucose; LDL, low density lipoprotein; HDL, high density lipoprotein; CABG, coronary artery bypass grafting; Cr, creatinine; CA cardiac arrest; SHR, stress hyperglycemia ratio; ECG, electrocardiogram; MI, miocardial infarction; AF, atrial fibrillation; ACCI, Charlson comorbidity index adjusted for age; CO2CP, CO2 binding force; Ca, calcium ACEI/ARB, angiotensin-converting enzyme inhibitors/angiotensin receptor blockers; NLR, ratio of neutrophil to lymphocyte counts; PLR, platelet to lymphocyte ratio; RDW, red blood cell distribution width; CRP, C-reactive protein; Hb, hemoglobin; MCV, mean cell volume; Na, sodium; PT, prothrombin time; DBP, diastolic blood pressure; K, potassium; LVEF, left ventricular ejection fraction; TC, total cholesterol; ITDM, insulin-dependent diabetes mellitus.

#### 3.4.3 Model validation and presentation methods

Among the studies validating predictive models, 13 studies (10, 11, 14, 16, 17–22, 24, 25, 27) utilized only internal validation methods, 2 study (15) employed only external validation, and 3 studies (12, 14, 23, 26) combined both internal and external validation. Regarding the presentation methods of the models, 6 studies (17–20, 25, 27) chose to display them through scoring systems, 2 studies (14, 26) opted for mobile websites, 2 studies (12, 23) used nomograms, while the remaining 8 studies (10, 11, 13, 15, 16, 21, 22, 24) did not specify the presentation methods of their models. For details, please refer to Table 5.

**Table 5:** Validation and presentation of prediction models.

Author	Verification method	Model presentation method
Jun Ke (10)/2022	Cross validation	No
Rong Li (11)/2023	Cross validation	No
Ashraf Abugroun (12)/2020	Internal verification, external verification	Nomogram
Bai Li (13)/2023	External verification	No
Sazzli Kasim (14)/2022	Cross validation	Mobile site
Claudio Parco (15)/2021	External verification	No
Konrad Pieszko (16)/2018	Cross validation	No
Raposeiras-Roubin S (17)/2012	Internal verification	Rating system
Raymond Bernardus (18)/2023	Internal verification	Rating system
Qiang Chen (19)/2022	Internal verification	Rating system
Rui Fu (20)/2018	Internal verification	Rating system
Rohan Khera (21)/2021	Internal verification	No
Joon-Myoung Kwon (22)/2019	Internal verification	No
He Lin (23)/2024	Cross validation, external verification	Nomogram
Konrad Pieszko (24)/2019	Cross validation	No
Chenxi Song (25)/2018	Internal verification	Rating system
Jingang Yang (26)/2024	Internal verification, external verification	Mobile site
Peng Ran (27)/2021	Internal verification	Rating system

### 3.5 Risk of bias and applicability assessment results

#### 3.5.1 Risk of bias domains

The overall risk of bias was high across all domains. In the participants domain, all studies (10–27) were identified as having a high risk of bias, primarily because the studies relied on retrospective cohort data, which depended on historical records. This led to issues such as missing data, recording errors, or inconsistencies. The selection of participants may not have been representative, and it was challenging to control for all confounding factors, resulting in potential information bias, selection bias, and confounding bias. In the predictors domain, 9 studies (12, 14, 19–22, 25–27) were assessed as having a high risk of bias, mainly due to the lack of uniformity in the definition and measurement of predictors. Data were derived from multi-center studies, where differences in patient characteristics, medical standards, and data collection methods across centers could introduce selection bias and performance bias. In the outcome domain, the same 9 studies (12, 14, 19–22, 25–27) were also considered to have a high risk of bias, primarily because of the multi-center nature of the data sources and inconsistencies in the definition and measurement of outcomes. The lack of standardization and the complexity of statistical analyses increased the likelihood of confounding bias. Two studies (16, 23) were rated as “unclear” because they did not specify the definition of outcomes. In the analysis domain, 1 study (14) was rated as having a low risk of bias, while 17 studies (10–13, 15–27) were rated as having a high risk of bias. Specific issues included: 6 studies (10, 11, 16, 19, 23, 24) had an events per variable (EPV) of <20; 2 studies (18, 20) converted continuous variables into binary categories; 3 studies (24, 26, 27) did not include all participants in the statistical analysis; 7 studies (12, 17, 19, 22, 25, 27) directly excluded missing values; 10 studies (10–12, 16, 19, 20, 23–25, 27) selected predictors based solely on univariate analysis, which was considered inappropriate; 9 studies (10, 16–18, 20, 22, 24, 25, 27) had incomplete evaluation of the models; 14 studies (10, 11, 13, 15–18, 20–25, 27) were overly optimistic in assessing model fit. These inappropriate experimental designs and data processing methods inevitably introduced varying degrees of bias risk. For details, please refer to Table 6.

**Table 6:** Risk of bias and applicability evaluation of included studies.

Include studies	Risk of bias				Applicability risk			Overall risk	
	Research object	Predictive factors	Final result	Analysis	Research object	Predictive factors	Final result	Risk of bias	Applicability
Jun Ke (10)/2022	-	+	+	-	+	+	+	-	+
Rong Li (11)/2023	-	+	+	-	+	+	+	-	+
Ashraf Abugroun (12)/2020	-	-	-	-	+	+	+	-	+
Bai Li (13)/2023	-	+	+	-	+	+	+	-	+
Sazzli Kasim (14)/2022	-	-	-	+	+	+	+	-	+
Claudio Parco (15)/2021	-	+	+	-	+	+	+	-	+
Konrad Pieszko (16)/2018	-	+	?	-	+	+	-	-	-
Raposeiras-Roubin S (17)/2012	-	+	+	-	+	+	+	-	+
Raymond Bernardus (18)/2023	-	+	+	-	+	+	-	-	-
Qiang Chen (19)/2022	-	-	-	-	+	+	-	-	-
Rui Fu (20)/2018	-	-	-	-	+	+	+	-	+
Rohan Khera (21)/2021	-	-	-	-	+	+	+	-	+
Joon-Myoung Kwon (22)/2019	-	-	-	-	+	+	+	-	+
He Lin (23)/2024	-	+	?	-	+	+	+	-	+
Konrad Pieszko (24)/2019	-	+	+	-	+	+	+	-	+
Chenxi Song (25)/2018	-	-	-	-	+	+	+	-	+
Jingang Yang (26)/2024	-	-	-	-	+	+	+	-	+
Peng Ran (27)/2021	-	-	-	-	+	+	+	-	+

+: Low risk of bias/high applicability; -: High risk of bias/low applicability; ?: unclear.

#### 3.5.2 Applicability assessment

In terms of applicability, 15 studies (10–15, 17, 20–27) demonstrated good overall applicability, while 3 studies (16, 18, 19) showed poor applicability. In the selection of study participants, all studies established inclusion and exclusion criteria that aligned with the principles of this review, demonstrating good applicability. Regarding the selection of predictors, all studies adhered to the inclusion principles of this review, also indicating good applicability. However, in the outcome domain, 3 studies (16, 18, 19) did not report specific definitions of the outcomes, resulting in poor applicability. For details, please refer to Table 6.

### 3.6 Key risk prediction models

In the field of clinical research, to more accurately grasp disease risks and improve diagnosis and treatment outcomes, it is crucial to sort out and evaluate various clinical risk prediction models. We first select the optimal model from each study by synthesizing factors such as predictive performance, stability, and applicability, and then screen out the most common models based on the frequency of occurrence of model construction methods. This initiative aims to promote direct comparison between different models, provide them with high-quality objects and a clear scope, so as to eliminate interference, enhance the reliability of results, and facilitate clinical decision-making and the development of the field. Details are shown in Table 7.

**Table 7:** Summary of key risk prediction models.

Author/year	Method/name	Key predictors (Top 3)	Key validation set AUC (95% CI)	Calibration metrics (brief)
Rong Li (11)/2023	XGBoost	HR, Age, MB	0.913 (0.910–0.916)	Calibration slope, calibration intercept, and Brier score
Bai Li (13)/2023	GRACE	Age, SBP, HR	0.926 (0.911–0.940)	Graphical analysis of risk model calibration/goodness-of-fit, HL
Raposeiras-Roubin S (17)/2012	GRACE	Age, HR, SBP	0.907 (0.889–0.924)	HL
Qiang Chen (19)/2022	GRACE + SHR	No importance analysis performed	0.814 (0.781–0.844)	Calibration curve, calibration slope, calibration intercept, Brier score
Rohan Khera (21)/2021	XGBoost	No importance analysis performed	0.898 (0.894–0.902)	Calibration slope, Brier score, shift table
Konrad Pieszko (24)/2019	XGBoost	No importance analysis performed	0.89 (not provided)	None
Jingang Yang (26)/2024	XGBoost	Age, LVEF, Killip	0.896 (0.884–0.909)	Calibration plot, HL
Author/year	External validation performed	Clinical application scenarios	Advantages	Limitations
Rong Li (11)/2023	No	Risk stratification for in-hospital death in ACS patients from 24 hours after admission to before discharge, especially suitable for assessment scenarios requiring combination of dynamic biomarkers and cardiac structural indicators.	XGBoost performance is significantly better than traditional models; incorporates dynamically changing indicators; excellent calibration effect; identifies new predictors	Single-center study; excludes patients with early death; low importance of ST-segment related indicators; lacks long-term prognosis assessment
Bai Li (13)/2023	Yes	All ACS patients (especially when comprehensive risk stratification is needed), recommended as the preferred model, suitable for assessment throughout hospitalization	Optimal comprehensive performance; applicable to all ACS subtypes; recommended by guidelines in multiple countries; high clinical recognition	Low proportion of Asians in the original development population; calculation requires laboratory data, slightly complex
Raposeiras-Roubin S (17)/2012	No	Early risk stratification of ACS patients after admission	High discrimination and high calibration; applicable to multiple populations; high clinical practicability; extremely high negative predictive value	High false positive rate in high-risk patients; does not include angiographic parameters
Qiang Chen (19)/2022	No	Suitable for risk stratification of in-hospital mortality in AMI patients	Has independent predictive value; good incremental predictive ability; clinical applicability of the combined model is higher than GRACE score alone; applicable to diabetic patients	Small sample size; retrospective cohort study; lack of external validation
Rohan Khera (21)/2021	No	Suitable for refined risk stratification of AMI patients after admission	Excellent calibration performance; strong risk reclassification ability; wide applicability in subgroups; no additional data required	Limited improvement in discriminative ability; uneven model performance; lack of external validation; variable limitations; lack of clinical tools
Konrad Pieszko (24)/2019	No	Applicable to in-hospital and long-term prediction of ACS patients	More accurate long-term prediction; relies on easily accessible indicators; integrates inflammatory mechanisms; easy for system integration	Slightly inferior in short-term prediction; small sample size; does not include clinical features; lack of external validation; single outcome
Jingang Yang (26)/2024	Yes	Suitable for early risk stratification of in-hospital mortality in STEMI patients after admission	Excellent prediction accuracy; strong interpretability; high flexibility; incorporates new key variables	Population limitations; variable restrictions; insufficient subgroup data

## 4 Discussion

### 4.1 Existing prediction models have clinical significance

Patients with ACS face a relatively high risk of in-hospital death. Constructing an accurate and effective risk prediction system and formulating intervention strategies in advance are of great significance for improving patient prognosis. In-hospital death risk prediction models can identify high-risk populations at an early stage, thus gaining time for clinical intervention. This review included 44 prediction models from 18 studies for analysis. The results showed that their AUC or C-index ranged from 0.79 to 0.96, indicating good discriminative ability and prediction accuracy, which enables accurate identification of patients at high risk of in-hospital death. Moreover, most models are presented in the form of scoring systems, which are easy to operate, understand, and use, meeting the needs of efficient clinical decision-making.

Age, systolic blood pressure, Killip classification, heart rate, and creatinine are frequently included predictors. Multiple studies (28–33) have confirmed that these factors are strongly associated with in-hospital death, serving as the core basis for risk modeling. It is worth noting that the RURUS SURYAWAN score proposed by scholars such as Suryawan IG (34) is designed for patients with acute myocardial infarction undergoing primary percutaneous coronary intervention. By quantifying clinical indicators to construct a scoring system, it achieves the stratification of 30-day death risk. From a practical perspective, it confirms the feasibility of the model construction path of “screening key factors—quantifying and assigning values—risk stratification” and also provides a reference for subgroup scenarios in the overall risk prediction of ACS.

In summary, in clinical practice, it is necessary to rely on existing prediction models, pay attention to the risk factors of in-hospital death, combine model scores with patients’ individual clinical characteristics, conduct dynamic assessments, and intervene in a timely manner to ensure patients’ in-hospital safety.

### 4.2 Data collection and processing impact prediction model performance

In this review, the in-hospital mortality rate of patients with ACS showed a significant variation (1.88%–12.3%), which is closely associated with inconsistent definitions of outcome indicators: 88% of the studies used “all-cause in-hospital mortality”, while 12% adopted “postoperative in-hospital mortality”. Such inconsistency severely impairs the cross-study comparability of models, leading to a lack of unified reference for prediction results and making it difficult to screen superior models. From the perspective of disease characteristics, ACS-related death is affected by multiple factors; focusing only on “postoperative in-hospital mortality” will miss non-surgical fatal events and fail to reflect the real risk. From the clinical practice perspective, it is unfavorable for evaluating the efficacy of conservative drug treatment and conducting objective comparisons of different treatment strategies. From the research value perspective, “all-cause in-hospital mortality” is more conducive to the promotion of research findings in different medical settings. Therefore, it is recommended that future studies on ACS in-hospital mortality prediction models uniformly take “all-cause in-hospital mortality” as the standardized outcome indicator, so as to facilitate global sharing of medical achievements and promote the advancement of ACS diagnosis and treatment.

All studies included in this review were retrospective cohort studies, involving extensive data collection and long-term follow-up. Such studies inherently face challenges with missing data, contributing to information bias. Additionally, the multicenter nature of the data sources led to inconsistent definitions and measurement standards for predictive factors, further increasing the risk of bias.

During the development of prediction models, the selection of predictive factors might not always be comprehensive, leading to potential information bias. For example, Konrad Pieszko and colleagues (24) utilized hospital electronic medical records for data collection and found that the data in medical records were often incomplete, complex, and disorganized. This introduced potential bias when extracting information for predictive factors. Particularly concerning was the presence of unstructured data stored in physicians’ notes, highlighting the importance of the expertise of the personnel designated to assess predictive factors. The performance of a model could vary significantly depending on whether experienced experts or inexperienced researchers handled this task.

Regarding sample size estimation, six studies (10, 11, 16, 19, 23, 24) did not meet the event per variable (EPV) principle, potentially leading to an overfitting risk in the models. Researchers must ensure a sufficient sample size to maintain model performance while recognizing that an excessively large sample size does not necessarily enhance model accuracy.

In terms of data preprocessing, 7 studies (12, 17, 19, 22, 25, 27) directly excluded missing data. This approach may bias the association between predictors and study outcomes, thereby constructing a biased model. Even if no bias occurs, it will still reduce the sample size and compromise information integrity, further decreasing the model's predictive accuracy. In the clinical data of ACS patients, variables such as laboratory indicators and comorbidities often have certain missing values. Simple exclusion or mean imputation can also lead to reduced sample size or data distortion, while multiple imputation can effectively retain sample information and reduce bias. To minimize the loss of valuable information during model development and evaluation, we should consider adopting advanced imputation techniques (e.g., multiple imputation) to appropriately account for the uncertainty of missing data, reduce bias, and improve model performance.

### 4.3 Variable selection affects prediction model performance

During the selection of predictive factors, 10 studies (10–12, 16, 19, 20, 23–25, 27) in this review used univariate analysis as the basis for variable selection. This approach might lead to improper selection of predictive factors because it overlooks interactions between variables and potential collinearity issues. When univariate modeling results in the omission of relevant variables, it introduces bias, causing overfitting and reducing the predictive accuracy of the model. Therefore, optimization during model development is crucial.

For instance, in the study by Jun Ke et al. (10), researchers initially performed univariate analysis to select the most appropriate variables for model development. To avoid overfitting and enhance model accuracy, they split the training dataset into a cross-validation scheme and adjusted the hyperparameters of each machine learning model to optimize cross-validation performance. The final model was then developed using the best hyperparameters to fit all training data.

Additionally, when selecting variables, it is essential not only to rely on statistical significance but also to consider potential confounding factors and other independent variables comprehensively. Ashraf Abugoun et al. (12) illustrated this approach while optimizing the modified CHA2DS2-VASc score. In their exploratory study, they found that hypertension and vascular disease had minimal impact on predicting mortality in ACS patients without a history of stroke. Conversely, low blood pressure and shock were associated with the highest mortality, while female gender contributed insignificantly to the model. As a result, they replaced hypertension with low blood pressure and shock, reduced the score for a history of stroke to 1 point, and removed the female gender variable.

Notably, contemporary model development needs to enhance the scientific rigor and transparency of feature selection. Among relevant approaches, penalized regression, cross-validation, and feature selection algorithms represent the best practices for screening predictive variables. High-dimensional data easily leads to model overfitting. LASSO compresses the coefficients of redundant features through L1 regularization, enabling simultaneous modeling and feature screening. Cross-validation dynamically tests the generalization ability of the model, avoiding evaluation bias caused by a single data partition. Feature selection algorithms reduce dimensionality and computational consumption in advance, and can also complement and optimize LASSO. These three approaches form a modeling loop, balancing accuracy, generalization, and interpretability, and serve as the key to addressing complex data. Therefore, we should adopt advanced techniques such as penalized regression methods and automatic feature selection with cross-validation. When screening predictors, we need to balance the correlation of variables and the generalization ability of the model, avoid model bias caused by the limitations of univariate analysis, reduce the risk of overfitting through technical approaches, and improve the rigor of model construction.

In summary, although existing prediction models are clinically instructive, whether for the development and validation of existing models or the reconstruction of new models, the data sources and the selection methods of predictors should be considered before construction. For example, prospective cohort studies with good data representativeness can be used, and predictors can be screened through literature review combined with multivariate analysis.

### 4.4 Application of artificial intelligence in in-hospital mortality risk prediction models for acute coronary syndrome

Currently, the construction methods for in-hospital death risk prediction models in ACS are relatively singular. Most studies adopt Logistic regression for modeling, while some studies attempt to break through traditional limitations through machine learning and deep learning technologies. For example, the team of Rong Li (11) applied the XGBoost algorithm, which showed higher accuracy than traditional logistic regression in identifying the risk of in-hospital death in ACS patients. Studies have demonstrated that machine learning is efficient and highly adaptive in processing large volumes of data, discovering complex patterns, and achieving accurate predictions (35); on this basis, deep learning can further automatically extract features, solve more complex problems, and realize high-precision prediction and classification (36). After comparing multiple methods, Sazzli Kasim et al. (14) confirmed that the deep learning model (SVM selected var) is more effective in predicting in-hospital mortality of ACS. These achievements fully confirm the great potential of AI technology in the field of risk prediction. However, from the perspective of clinical practice, traditional scoring systems are still applied more frequently in real-world settings.

In this review, multiple studies (13, 16, 17, 19, 25, 27) indicate that the GRACE model still performs excellently in various aspects. It remains a reliable tool for ACS risk prediction in the foreseeable future and is currently the most suitable model for routine clinical use. This score was developed based on large-scale, unbiased multicenter registry data and validated by external datasets, thus showing excellent performance when applied to the general population. However, its prediction accuracy for patients undergoing PCI is suboptimal. Therefore, there is a need for updated risk scores adapted to current clinical practices to supplement the application of existing scoring systems.

Among the 8 studies (10, 11, 14, 16, 21, 22, 24, 26) included in this review, machine learning and deep learning technologies were applied either independently or in combination, and the constructed models showed excellent performance in predictive ability. Among them, only the model constructed by scholars such as Sazzli Kasim (14) has been integrated into routine clinical diagnosis and treatment processes. This model has been deployed on a risk calculator within the hospital's internal network, but the network is not open to the public as the research is still in the testing phase. Other machine learning-based methods have not yet been fully validated in clinical integration, and most remain in the stage of research and small-scale validation. The core obstacles lie in the universality of validation, interpretability, and compatibility with clinical workflows. For interpretability assessment, only 3 studies (11, 14, 26) employed the SHAP value method. The remaining studies merely reported model performance without clarifying the logic underlying predictive outcomes. Clinicians, however, need to understand this logic to trust the model; unexplainable “black-box models” may cause confusion in clinical decision-making, highlighting a severe lack of interpretability. In terms of reproducibility, only 3 studies (14, 24, 26) made model codes or detailed parameter settings publicly available. For the rest, incomplete methodological reporting rendered the model construction process irreproducible. In contrast, regression-based models exhibit significantly higher reproducibility due to their transparent parameters and simple calculation. Regarding clinical integration, only 1 study (14) conducted clinical applicability testing; the remaining studies only achieved performance validation at the data level. Clinical decision-making for ACS requires models to be “fast and convenient,” yet most current AI models fail to meet practical clinical needs, as they are time-consuming for computation and require professional software support. It is thus evident that compared with regression-based models, current AI models in the ACS field have obvious disadvantages in “interpretability, reproducibility, and clinical integration.” The translation of AI technology from research to clinical application still requires addressing key issues.

Firstly, the adaptability to clinical scenarios needs to be clarified. Traditional scoring systems, due to their simplicity of operation and mature clinical application, still have advantages in primary medical institutions or rapid emergency assessment. Although AI models have higher prediction accuracy, their operational complexity and the difficulty in interpreting results may affect clinical acceptance. The SPADAFORA L team (37) included 23,270 ACS patients and found that the impact of in-hospital bleeding (IHB) on 1-year prognosis varies among subgroups such as age, gender, and treatment pathways. This suggests that different models need further comparison in specific scenarios. For example, regarding the precise stratification of complex cases, whether the performance advantages of AI models can cover their application costs still requires more practical verification.

Secondly, the value of clinical intervention needs to be deepened. The SPADAFORA L team (37) also revealed that IHB, as one of the markers of the severity of ACS patients’ condition, suggests that risk prediction models should expand their dimensions, not limited to identifying death risks, but also include indicators such as bleeding risk and prognostic changes after intervention. However, existing AI models mostly remain in the stage of risk stratification and have not fully explored their guiding role in clinical decision-making. For example, whether dynamic risk assessment can be used to adjust the intensity of antithrombotic therapy, optimize monitoring frequency, and whether they can more effectively reduce the incidence of adverse events and improve patients’ long-term prognosis compared with traditional models, these still need in-depth research combined with clinical practice.

In general, artificial intelligence technology has provided new tools for ACS risk prediction and shown great potential. However, the full realization of its clinical value needs to focus on the verification of scenario adaptability and the exploration of intervention pathways, focusing on the closed-loop verification of “model-clinical scenario-patient outcome”. Through more real-world studies, the technology can be promoted from “accurate prediction” to “clinical practicality”, ultimately achieving effective supplementation and optimization of traditional models.

### 4.5 The applicability of prediction models requires further validation

Model validation is a critical step in assessing the performance and generalizability of prediction models, involving both internal and external validation. Internal validation estimates model performance by training and evaluating the model on the same dataset, which helps identify whether the model is overfitting or underfitting. In contrast, external validation evaluates the model on an independent dataset to assess its generalizability and extrapolation capability.

In this review, 13 studies (10, 11, 14, 16, 17–22, 24, 25, 27) conducted only internal validation, while 2 study (13, 15) performed external validation exclusively. Therefore, 72% of ACS in-hospital mortality prediction models may be overfitted due to the lack of external validation, resulting in insufficient clinical generalization. Unless a model undergoes external validation across multiple centers and diverse populations (e.g., cross-regional and cross-ethnic cohorts), it is not recommended for direct use in clinical decision-making; further validation is still required to support its clinical application. Many researchers acknowledged the limitation of lacking external validation and expressed concerns about the model's applicability to different regional populations.

For example, in the study by Peng Ran et al. (27), although the model was developed using a large dataset, it was limited to Chinese patients. The authors highlighted the need for further research to verify the model's performance in other populations and emphasized the necessity of external validation before widespread clinical adoption.

Overall, most studies on in-hospital mortality risk prediction models for ACS are single-center studies, lacking consideration for differences in applicability across diverse cultural and geographical environments.

Therefore, when constructing in-hospital mortality risk prediction models for acute coronary syndrome, researchers should integrate both internal and external validation. Techniques such as cross-validation, bootstrap resampling, and the “internal-external” approach can be employed for internal validation, while temporal validation, spatial validation, and domain validation methods can be utilized for external validation. Additionally, conducting multi-center studies can significantly enhance the generalizability of the prediction models.

### 4.6 The presentation and reporting of prediction models need further standardization

The reporting of prediction model results should adhere to the Transparent Reporting of a Multivariate Prediction Model for Individual Prognosis or Diagnosis (TRIPOD) statement, ensuring that the report includes a complete model equation to enable reproducibility and independent external validation studies. Unfortunately, all studies included in this review lack transparency in their construction processes, with information gaps that affect the quality assessment of the literature. When evaluating prediction models, model calibration is a core indicator for measuring model reliability, whose importance is equivalent to or even greater than discriminative ability in clinical practice. Therefore, in addition to these two core indicators, comprehensive evaluation should be conducted from multiple aspects such as overall performance, reclassification, and clinical utility to improve the assessment of model performance. For example, consideration should be given to indicators including the model's sensitivity, specificity, accuracy, as well as the Hosmer-Leme show test and calibration curve that directly reflect calibration performance, while combining metrics like Decision Curve Analysis (DCA) and clinical impact curve. Notably, although DCA is highly valuable for evaluating the clinical utility of clinical prediction models, its adoption in practical research remains low. Three reasons account for this: first, traditional studies focus more on model predictive accuracy and insufficiently emphasize “clinical utility,” with inertial thinking leading to DCA being overlooked; second, DCA is not a universal indicator—it only applies to models for which “interventions are needed after outcome prediction,” resulting in limited application scope; third, compared with easily calculable indicators such as AUC and calibration curves, DCA is more complex to operate, requiring higher data standards and relying on professional programming software, which raises the threshold for researchers to use it.

Furthermore, there are challenges in applying machine learning and deep learning to model construction while adhering to TRIPOD. Since machine learning and deep learning models are often regarded as “black boxes,” their internal decision-making processes and interpretability of feature impacts are poor, which contradicts the transparency required by the TRIPOD statement. The TRIPOD statement does not provide detailed reporting guidelines for feature selection of input variables and feature engineering that improves and transforms raw data, leading to deficiencies in reporting—particularly the potential neglect of systematic assessment and reporting of calibration, which is a critical prerequisite for the application of models in clinical decision-making. When applying machine learning or deep learning algorithms, researchers should select appropriate visualization and interpretation tools to demonstrate the impact of each variable on outcomes, while ensuring the practical significance of model-predicted probabilities through rigorous calibration validation. For instance, when constructing a deep learning model, Rui Fu (20) and colleagues, despite being able to fit the model through individual weights, still found it difficult to interpret the deep learning model using methods such as variable importance or risk score-based decision-making. This highlights the need for further exploration in the field of interpretable deep learning, with optimization and validation of calibration as one of its core objectives. When using machine learning models, Jingang Yang (26) and colleagues utilized SHAP (SHapley Additive exPlanations) to explain how the predicted risk for individual patients is determined, revealed the complex relationships between predictors and outcomes embedded in the XGBoost model, and combined this with calibration assessment—greatly enhancing the clinical credibility of the model.

Therefore, when constructing risk prediction models, scholars should strictly follow the requirements of the TRIPOD document, attach importance to and standardize the assessment and reporting of model calibration, comprehensively improve the transparency of the construction process, and further optimize the application of artificial intelligence technology in prediction model construction.

## 5 Limitations of the study

This systematic review has several limitations: (1) The review included only Chinese and English literature and searched only five databases, potentially leading to literature omissions. (2) The included studies were predominantly conducted in Chinese regions, which may limit the generalizability of the findings to Western countries and other diverse populations. (3) This study only included Chinese and English literature, which may introduce language bias. Additionally, 50% of the study populations were Chinese, leading to an overrepresentation of models developed for the Chinese population and insufficient coverage of models for other regions. Consequently, the conclusions have low applicability to non-Chinese populations.

## 6 Conclusion

The construction of in-hospital mortality risk prediction models for acute coronary syndrome (ACS) is currently in a phase of rapid development. While many models demonstrate good predictive ability, there remain significant gaps in data analysis and processing methods. Many studies did not adhere to the TRIPOD reporting guidelines, lacked external validation, and were predominantly single-center studies, resulting in a high overall risk of bias and limited generalizability.

Looking forward, the development of ACS in-hospital mortality risk prediction models should follow the PROBAST standards to create models with strong predictive performance and broad applicability. Rigorous adherence to reporting and validation protocols will enhance the clinical utility and reliability of these models.

## Data Availability

The raw data supporting the conclusions of this article will be made available by the authors, without undue reservation.
